# Emergence of *Staphylococcus lugdunensis* as a Cause of Urinary Tract Infection: Results of the Routine Use of MALDI-TOF MS

**DOI:** 10.3390/microorganisms8030381

**Published:** 2020-03-09

**Authors:** Kelvin H. Y. Chiu, Rex P. K. Lam, Elaine Chan, Susanna K. P. Lau, Patrick C. Y. Woo

**Affiliations:** 1 Department of Microbiology, Li Ka Shing Faculty of Medicine, The University of Hong Kong, Hong Kong, China; hychiu09@gmail.com (K.H.Y.C.); elaineee@gmail.com (E.C.); 2 Emergency Medicine Unit, Li Ka Shing Faculty of Medicine, The University of Hong Kong, Hong Kong, China; lampkrex@hku.hk; 3 State Key Laboratory of Emerging Infectious Diseases, The University of Hong Kong, Hong Kong, China; 4 Collaborative Innovation Center for Diagnosis and Treatment of Infectious Diseases, The University of Hong Kong, Hong Kong, China

**Keywords:** *Staphylococcus lugdunensis*, urinary tract infection, MALDI-TOF MS

## Abstract

We analyzed the incidence and the clinical and laboratory characteristics of *Staphylococcus lugdunensis* urinary tract infections (UTIs) during a 10-year period (2009–2018) and compared them with those of *Staphylococcus saprophyticus* UTIs. A total of 38 and 162 episodes of *S. lugdunensis* and *S. saprophyticus* UTIs were observed. The number of *S. saprophyticus* UTIs was stable throughout the 10 years, whereas there was an obvious surge in the apparent number of *S. lugdunensis* UTIs since 2014, coinciding with the commencement of a routine use of MALDI-TOF MS. Univariate analysis showed that male sex (*p* < 0.001), advanced age (*p* < 0.001), hospital-acquired infections, (*p* < 0.001), upper UTI (*p* < 0.005), polymicrobial infections (*p* < 0.05), hypertension (*p* < 0.001), solid-organ malignancies (*p* < 0.001), renal stones (*p* < 0.001), urinary stricture (*p* < 0.05), vesicoureteral reflux (*p* < 0.001), and presence of a urinary catheter (*p* < 0.001) were significantly associated with *S. lugdunensis* UTI. Multivariable analysis revealed that *S. lugdunensis* UTI was associated with male sex (OR = 6.08, *p* < 0.05), solid-organ malignancies (OR = 12.27, *p* < 0.01), and urological system abnormalities (OR = 7.44, *p* < 0.05). There were significant differences in the patient population affected and predisposing factors between *S. lugdunensis* and *S. saprophyticus* UTIs.

## 1. Introduction

Traditionally, staphylococci have been classified phenotypically by the presence or absence of coagulase. This method is also used to identify individual staphylococcus strains such as coagulase-positive *Staphylococcus aureus* or coagulase-negative staphylococci. Among the coagulase-negative staphylococci, *Staphylococcus saprophyticus*, identified phenotypically by its resistance to novobiocin, is the second most important cause of community-acquired urinary tract infection (UTI) in sexually active young females [1]. Apart from *S. saprophyticus*, other coagulase-negative staphylococci isolated from the urinary tract were often not further identified to the species level, and therefore their roles in UTI cannot be ascertained. In recent years, another coagulase-negative staphylococcus, *Staphylococcus lugdunensis*, has emerged as a cause of invasive bacterial infections, such as infective endocarditis and skin and soft tissue infections [2]. It is of note that both *S. lugdunensis* and *S. saprophyticus* reside around the genitourinary tract, with *S. lugdunensis* colonizing the pelvic, perineum, and groin region, and *S. saprophyticus* colonizing the rectum and genitourinary tract [3]. Considering their similar localization in the human body as well as their important virulence properties, we hypothesized that *S. lugdunensis* is a previously underestimated pathogen of the urinary tract. Although case reports and small case series of *S. lugdunensis* UTI have been described [4,5,6], there has been no systematic analysis of the clinical and laboratory characteristics on this clinical entity.

Matrix-assisted laser desorption ionization time-of-flight mass spectrometry (MALDI-TOF MS) has recently emerged as a revolutionary technique for the identification of bacterial pathogens, yielding rapid, accurate, and highly reproducible results at a lower price than any other methods routinely used in clinical laboratories [7]. The methodology is easy to follow, requires only a minimal amount of bacteria for the analysis, and provides results within minutes. Therefore, it has now been integrated into many clinical laboratories and is useful for the identification of different groups of medically important bacteria [8,9,10]. Since the start of the routine use of MALDI-TOF MS in our clinical microbiology laboratory in January 2014, we have observed an unprecedented surge of *S. lugdunensis* isolates identified in urine samples of our patients. In this study, we describe the apparent emergence and the clinical and laboratory characteristics of patients with UTIs caused by *S. lugdunensis* in a 10-year period and compare them with those of *S. saprophyticus* in the same study period.

## 2. Materials and Methods

### 2.1. Ethical Statement

This study was approved by the Institutional Review Board of The University of Hong Kong/Hospital Authority (UW16-365, approved 22-07-2016).

### 2.2. Patients

**Figure 1:**
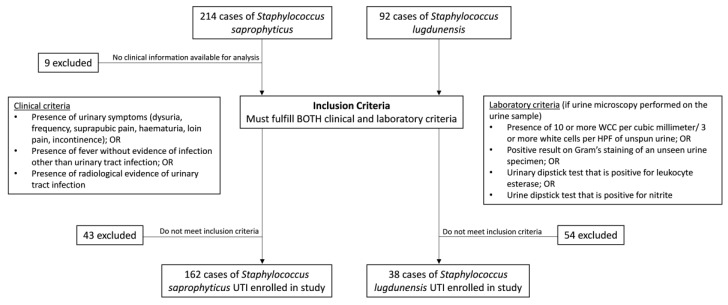
Enrollment flowchart of urinary tract infection (UTI) cases caused by *Staphylococcus saprophyticus* and *Staphylococcus lugdunensis* in 2009–2018.

All patients whose urine samples were found positive to *S. lugdunensis* and *S. saprophyticus* during a 10-year period (2009–2018) in hospitals in Hong Kong West Cluster—including Queen Mary Hospital, Tung Wah hospital, Grantham Hospital, and Fung Yiu King Hospital of Hong Kong—were included in the study. The medical records of the patients were retrieved for analysis. Those episodes of illness that met the definition of UTI given below were included for further analysis (Figure 1).

### 2.3. Microbiological Methods

Before January 2014, all staphylococci were first tested with Staphaurex plus latex agglutination test (Thermo Scientific). For all coagulase-negative staphylococci, the novobiocin disk diffusion test was used to identify *S. saprophyticus* (novobiocin-resistant). All novobiocin-sensitive coagulase-negative staphylococci were discarded, unless the microbiologist had requested their full identification. After January 2014, all staphylococci were preliminarily identified by MALDI-TOF MS. When MALDI-TOF MS results showed the presence of *S. lugdunensis*, bacterial identity was further confirmed by positive Staphaurex plus latex agglutination test and negative tube coagulase test. When the MALDI-TOF MS results showed the presence of *S. saprophyticus*, bacterial identity was further confirmed by negative Staphaurex plus latex agglutination test and resistance to novobiocin.

### 2.4. Definitions

UTI was defined based on the fulfillment of both clinical and laboratory criteria. Clinical identification included the fulfillment of at least one of the following criteria: presence of urinary symptoms (dysuria, urinary frequency, suprapubic pain, gross hematuria, loin pain, urinary incontinence); fever without other culture result suggestive of infection other than UTI; and radiological evidence of UTI. Laboratory criteria included the isolation at least 10^4^ colony-forming unit (cfu) per mL of urine of the target microorganism and, if urine microscopy or biochemical tests were performed, the presence of pyuria, determined on the basis of either one of the following: 10 or more white blood cells per mL, 3 or more white blood cells per high-power field of unspun urine, presence of bacteria identified by direct microscopy, positivity for leukocyte esterase or nitrite in the urine dipstick test.

### 2.5. Statistical Analysis

Categorical variables were analyzed by Fisher’s exact test or Chi-square test, and continuous variables were analyzed by Mann–Whitney U test using SPSS version 24.0 (IBM Corp., Armonk, NY, USA). A *p* value <0.05 was considered statistically significant. Variables that were considered as statistically significant in univariate analysis were subjected to multivariable analysis by binomial logistic regression.

## 3. Results

### 3.1. Emergence of UTI Caused by S. lugdunensis

**Figure 2:**
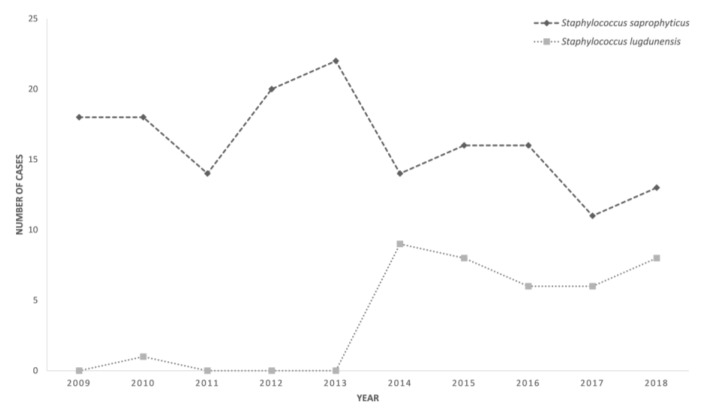
Cases of *S. saprophyticus*- and *S. lugdunensis*-associated urinary tract infection in 2009–2018.

From January 2009 to December 2018, a total of 306 non-duplicated *S. lugdunensis* and *S. saprophyticus* isolates were recovered from the urine samples of 289 patients. Two hundred (from 190 patients) of the 306 episodes fulfilled the diagnostic criteria of UTI and were subjected to further analysis. Among these 200 episodes, 38 were *S. lugdunensis* and 162 were *S. saprophyticus* infections. The number of *S. saprophyticus* UTI episodes was stable throughout the 10 years, whereas there was an obvious surge in the apparent number of *S. lugdunensis* UTI episodes since 2014, coinciding with the commencement of the use of MALDI-TOF MS in our clinical microbiology laboratory (Figure 2). In fact, there was only one *S. lugdunensis* UTI episode from 2009 to 2013, whereas there were 38 *S. lugdunensis* UTI episodes from 2014 to 2018.

### 3.2. Characteristics of Patients with S. lugdunensis UTI

**Figure 3:**
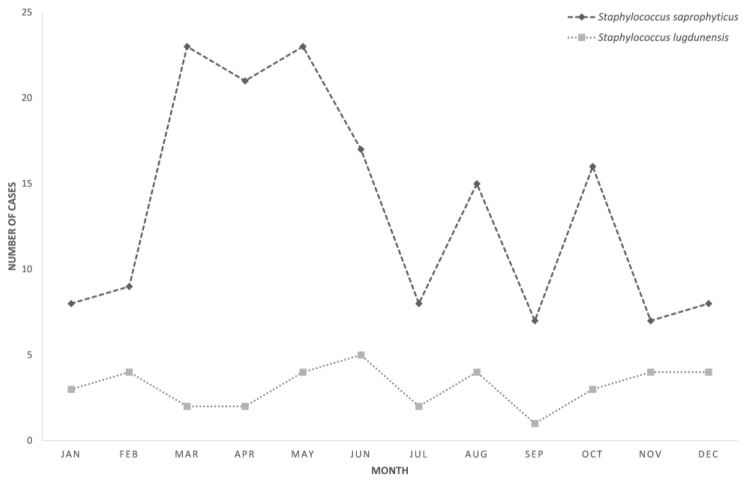
Cases of *S. saprophyticus*- and *S. lugdunensis*-associated urinary tract infection recorded by month of specimen collection (2009 – 2018).

Patients with *S. lugdunensis* UTI were predominantly male (71.1%), whereas only 3.1% of those with *S. saprophyticus* UTI were male (*p* < 0.001). The median age of patients with *S. lugdunensis* UTI was 59 (range 27–90), significantly higher than that of patients with *S. saprophyticus* UTI (median 29, range 4–90) (*p* < 0.001) (Table 1). There was no obvious seasonal variation for *S. lugdunensis* UTI (Figure 3). In addition, 15.8% of *S. lugdunensis* UTIs were hospital-acquired infections, whereas only 1.9% of *S. saprophyticus* UTIs were hospital-acquired (*p* < 0.001); 15.8% of *S. lugdunensis* UTIs were upper urinary tract infections, whereas only 2.5% of *S. saprophyticus* UTIs were upper urinary tract infections (*p* < 0.005). None of the patients with *S. lugdunensis* UTI developed *S. lugdunensis* bacteremia, and none of them died during the episode of UTI.

Patients with *S. lugdunensis* UTI were significantly more likely to have co-existing bacteria in the urine than those with *S. saprophyticus* UTI (26.3% and 11.1%, respectively, *p* < 0.05). The three commonest co-existing organisms isolated from the urine of *S. lugdunensis* UTI patients were *Enterococcus* species (40.0%), coagulase-negative staphylococci other than *S. saprophyticus* (30.0%), and *Escherichia coli* (20.0%). The three commonest co-existing organisms isolated from the urine of *S. saprophyticus* UTI patients were *E. coli* (44.4%), *Corynebacterium* species (11.1%), and *Citrobacter* species (11.1%).

As for the association with general underlying diseases, the incidences of hypertension and solid-organ malignancies in patients with *S. lugdunensis* UTI (39.5% and 44.7%, respectively) were significantly higher than those in patients with *S. saprophyticus* UTI (4.9% and 1.2%, respectively) (*p* < 0.001 and *p* < 0.001, respectively). As for local predisposing factors and urological system abnormalities, the incidences of renal stones, urinary stricture, vesicoureteral reflux, and presence of a urinary catheter in patients with *S. lugdunensis* UTI (23.7%, 7.9%, 7.9%, and 34.2%, respectively) were significantly higher than those in patients with *S. saprophyticus* UTI (3.1%, 0.6%, 0% and 1.9%, respectively) (*p* < 0.001, *p* < 0.05, *p* < 0.001 and *p* < 0.001, respectively).

Multivariable analysis using binomial logistic regression revealed that *S. lugdunensis* UTI was associated with male sex (odds ratio [OR] = 6.08, *p* < 0.05), solid-organ malignancies (OR = 12.27, *p* < 0.01), and urological system abnormalities (OR = 7.44, *p* < 0.05) (Table 2).

### 3.3. Recurrent S. lugdunensis UTI

Two of the 38 patients had recurrence of *S. lugdunensis* UTI. The first patient was a 69-year-old Chinese man with a carcinoma of the bladder diagnosed six years before, who was treated with transurethral resection of the bladder tumor and intravesical bacillus Calmette–Guerin (BCG) therapy. He presented with dysuria that had been ongoing for two days, with turbid urine. *S. lugdunensis* (susceptible to ciprofloxacin, erythromycin, methicillin, nitrofurantoin, cotrimoxazole, gentamicin, and vancomycin) was recovered from his urine. Subsequent investigation revealed right vesico–ureteric junction narrowing, with vesicoureteral reflux. He was given oral amoxicillin–clavulanate for one week. However, the symptoms recurred 11 days after stopping the antibiotic. At that time, *S. lugdunensis* with a different antibiotic susceptibility profile (susceptible to ciprofloxacin, erythromycin, methicillin, nitrofurantoin, cotrimoxazole, and vancomycin but resistant to gentamicin) was recovered from his urine. He was given oral amoxicillin–clavulanate again for one week, and there were no more recurrences of *S. lugdunensis* UTI.

The second patient was a 53-year-old Chinese man with underlying left renal stone and right ureteric stone, with right-sided hydronephrosis. Extracorporeal shock wave lithotripsy and subsequent ureterorenoscopic lithotripsy was performed one year before presentation. He initially presented with bilateral loin discomfort. Urine microscopy showed abundant white blood cells, and urine culture recovered a pure growth of >10^5^ cfu/mL *S. lugdunensis* (susceptible to nitrofurantoin and cotrimoxazole but resistant to methicillin and gentamicin). Antibiotics treatment was not administered, but the patient’s symptoms gradually subsided. Three months later, extracorporeal shock wave lithotripsy was repeated, and the patient developed fever and bilateral loin discomfort four days later. Computed tomography showed a renal stone at the left pelvo–ureteric junction, with hydronephrosis. A left JJ stent was inserted. Microscopic examination of the ureteric urine showed abundant leukocytes, and urine culture recovered *S. lugdunensis* with a different antibiotic susceptibility profile (susceptible to methicillin, nitrofurantoin, and cotrimoxazole but resistant to gentamicin). He was given intravenous and then oral cefuroxime for one week, which stopped the recurrence of *S. lugdunensis* UTI.

## 4. Discussion

This is the first study analyzing the clinical and laboratory characteristics of *S. lugdunensis* UTI systematically. Upon analysis of all patients with *S. lugdunensis* UTI in the last 10 years, it was observed that there was an apparent emergence of UTI caused by *S. lugdunensis* since 2014 (Figure 2). From 2009 to 2014, when *S. lugdunensis* was identified by positive Staphaurex plus latex agglutination test and negative tube coagulase test, only one case of *S. lugdunensis* UTI was recorded. This was because *S. lugdunensis* was not recognized as an important urinary tract pathogen, and coagulase-negative staphylococci that were not *S. saprophyticus* were not further identified to the species level in the past. The marked increase in the detection of *S. lugdunensis* isolates in urine samples since 2014 coincided with the commencement of using MALDI-TOF MS for rapid bacterial identification in our clinical microbiology laboratory. The use of MALDI-TOF MS has facilitated the identification of a number of bacteria in our laboratory, such as *Burkholderia pseudomallei*, *Laribacter hongkongensis*, and *Tsukamurella* species [8,9,10]. This method not only increases the number of cases diagnosed but also shortens the time to diagnosis. In the past, when conventional methods were used for bacteria identification, it took at least 24 h to confirm the identities of most bacteria, whereas MALDI-TOF MS only requires a few minutes. This improvement in the laboratory diagnosis of *S. lugdunensis* UTI gave us a good opportunity to analyze the epidemiology, predisposing factors, clinical spectrum, and infection outcome of this distinct disease entity. In contrast to *S. lugdunensis*, there have been no changes in the incidence of *S. saprophyticus* UTI in the last 10 years, though MALDI-TOF MS can shorten the time of identification of this bacterium as well.

There were significant differences in the patient population affected and the predisposing factors between *S. lugdunensis* and *S. saprophyticus* UTIs. Similar to what was described in the literature [11], the present cohort of patients with *S. saprophyticus* UTI were mainly young and healthy females with lower UTI. On the other hand, patients with *S. lugdunensis* UTI were predominantly males and much older (Table 1). A significant proportion of *S. lugdunensis* UTI patients had underlying diseases, most importantly solid-organ malignancies and urological abnormalities (Table 1). Solid-organ malignancies associated with *S. lugdunensis* UTI, 53.8% involved the genitourinary system [bladder (*n* = 4), prostate (*n* = 4), kidney (*n* = 1), ovary (*n* = 1), vagina (*n* = 1)], whereas the most common urological abnormalities included the presence of renal stones, urinary stricture, and vesicoureteral reflux. It is of note that the two patients in this cohort with recurrent *S. lugdunensis* UTI had underlying urological abnormalities: carcinoma of the bladder and vesicoureteral reflux in the first patient, and presence of renal stones in the second patient. These urological abnormalities enhance the colonization of pathogenic bacteria and increase the difficulty in clearing them. After all, although patients with *S. lugdunensis* were older with underlying diseases, none of them developed bacteremia or died during the corresponding episodes of UTI, indicating tha *S. lugdunensis* UTI is still a relatively benign and readily treatable infection.

The pathogenesis of *S. lugdunensis* UTI remains to be determined. *S. saprophyticus* is primarily a pathogen of the urinary tract, and the factors involved in the mediation of UTI caused by *S. saprophyticus* are relatively well studied. *S. saprophyticus* possesses various types of adhesins such as hemagglutinins, autolysin Ass, and surface-associated lipases such as Ssp, which facilitate effective bacterial adherence to fibronectin and fibrinogen and, hence, colonization of the uroepithelium [12,13,14]. It can also form a bacterial biofilm, which confers an anti-phagocytic and antimicrobial-resistance phenotype and increases the virulence of the bacteria [15]. Furthermore, *S. saprophyticus* is urease-positive, which is important for bacterial colonization and invasion. Essentially, urease catalyzes the hydrolysis of urea to carbon dioxide and ammonia, which results in an increase in urine pH and the production of calcium and magnesium phosphate crystals in the urine. This accumulation of ammonia is toxic to uroepithelial cells and causes direct tissue damage [16]. As for *S. lugdunensis*, its virulence properties were often studied in the context of other infections that it can cause, such as infective endocarditis and catheter-related bacteremia [17]. *S. lugdunensis* does not produce urease but does encode a δ-like hemolysin that shares phenotypic properties with the *S. aureus* delta-toxin [18]. The *S. aureus*-produced delta-toxin can attach non-specifically to the cytoplasmic membrane of a cell and form short-lived membrane-damaging pores which cause cell lysis and subsequent cell death. In addition, *S. lugdunensis* encodes the staphylococcal *agr* locus, a quorum-sensing locus that regulates a number of virulence genes such as enterotoxins and hemolysins [19], and a *S. lugdunensis* synergistic hemolysin (*slush*) locus, which encodes three highly similar 43-amino-acid hemolytic peptides with delta-toxin-like activity [20]. These hemolytic proteins may potentially contribute to the invasion of the bacteria and cause tissue damage as observed for urease activity. Moreover, *S. lugdunensis* encodes host surface adherence proteins such as the multifunctional autolysin/adhesin AtlL, which binds to the extracellular matrix, to plasma proteins such as fibronectin and fibrinogen, and to human endothelial cells [21]. Mutation of AtlL also result in a significantly reduced ability of the bacteria to form biofilm. We speculate that these virulence factors may mediate *S. lugdunensis* UTI in a similar way as that observed for *S. saprophyticus*, via adherence and colonization of the uroepithelium and biofilm formation, particularly in the presence of urinary catheters on which biofilm can easily form. Underlying urological abnormalities that lead to stagnation of urine are important for *S. lugdunensis* multiplication. Further studies are required to delineate which of these virulence factors are particularly important for the pathogenesis of *S. lugdunensis* UTI.

## 5. Conclusions

An apparent surge in the number of *S. lugdunensis* UTI was observed since 2014, which coincided with the start of the routine use of MALDI-TOF MS in our clinical microbiology laboratory. In comparison with UTI caused by *S. saprophyticus*, significant differences in the patient population affected and predisposing factors were found for *S. lugdunensis* UTI.

## Figures and Tables

**Table 1:** Clinical characteristics of *S. lugdunensis* and *S. saprophyticus* urinary tract infection.

Characteristics	*S. lugdunensis* UTI (*n* = 38)	*S. saprophyticus* UTI (*n* = 162)	*p* Value
Sex (male)	27 (71.1)	5 (3.1)	< 0.001
Age (median, range)	59.0 (27–90)	29.0 (4–90)	< 0.001 ^1^
Hospital-acquired	6 (15.8)	3 (1.9)	0.002
Upper UTI	6 (15.8)	4 (2.5)	0.004
Polymicrobial infection	10 (26.3)	18 (11.1)	0.015
Local predisposing factors and urological system abnormalities			
Renal stone	9 (23.7)	5 (3.1)	< 0.001
Urinary stricture	3 (7.9)	1 (0.6)	0.022
Vesicoureteral reflux	3 (7.9)	0 (0.0)	0.006
Renal transplantation	1 (2.6)	0 (0.0)	0.190
Urinary catheter	13 (34.2)	3 (1.9)	< 0.001
Other underlying illness			
Hypertension	15 (39.5)	8 (4.9)	< 0.001
Solid-organ malignancy	17 (44.7)	2 (1.2)	< 0.001
Hematological malignancy	1 (2.6)	0 (0.0)	0.190
Diabetes mellitus	3 (7.9)	6 (3.7)	0.376
Immunodeficiency	3 (7.9)	7 (4.3)	0.405
Cerebrovascular accident	2 (5.3)	2 (1.2)	0.164
End-stage renal failure	2 (5.3)	1 (0.6)	0.093

Data are presented as No. (%) unless otherwise indicated. All statistical analyses were carried out by the Fisher’s exact test unless otherwise indicated. ^1^ Calculated by Mann–Whitney U test.

**Table 2:** Results of a multivariable analysis of the clinical characteristics associated with *S. lugdunensis* and *S. saprophyticus* urinary tract infection.

	Multivariable Analysis		
Characteristics	OR	95% CI	*p* Value
Sex (male)	6.08	1.26–29.43	0.025
Age (median, range)	1.03	0.99–1.07	0.115
Hospital acquired	3.72	0.52–26.82	0.193
Upper UTI	6.69	0.46–98.18	0.165
Local predisposing factors and urological system abnormalities ^1^	7.44	1.52–36.37	0.013
Hypertension	3.62	0.60–21.70	0.159
Solid-organ malignancy	12.27	1.800–83.69	0.010

All statistical analyses were carried out by the Fisher’s exact test unless otherwise indicated. CI, confidence interval; OR, odds ratio. ^1^ Includes incidence of renal stones, urinary stricture, vesicoureteral reflux, renal transplantation, and presence of a urinary catheter.
